# The effects of COVID-19 event strength on job burnout among primary medical staff

**DOI:** 10.1186/s12913-023-10209-z

**Published:** 2023-11-06

**Authors:** Xinru Li, Yiwen Song, Bingqin Hu, Yitong Chen, Peiyao Cui, Yifang Liang, Xin He, Guofeng Yang, Jinghua Li

**Affiliations:** School of Public Health, Jilin University, NO.1163 Xinmin Street, Changchun, Jilin Province China

**Keywords:** COVID-19, Event system theory, Job burnout, Job stress, Work engagement, Primary health care

## Abstract

**Background:**

As a global pandemic, The Corona Virus Disease 2019 (COVID-19) has brought significant challenges to the primary health care (PHC) system. Health professionals are constantly affected by the pandemic’s harmful impact on their mental health and are at significant risk of job burnout. Therefore, it is essential to gain a comprehensive understanding of how their burnout was affected. The study aimed to examine the relationship between COVID-19 event strength and job burnout among PHC providers and to explore the single mediating effect of job stress and work engagement and the chain mediating effect of these two variables on this relationship.

**Methods:**

Multilevel stratified convenience sampling method was used to recruit 1148 primary medical staff from 48 PHC institutions in Jilin Province, China. All participants completed questionnaires regarding sociodemographic characteristics, COVID-19 event strength, job stress, work engagement, and job burnout. The chain mediation model was analyzed using SPSS PROCESS 3.5 Macro Model 6.

**Results:**

COVID-19 event strength not only positively predicted job burnout, but also indirectly influenced job burnout through the mediation of job stress and work engagement, thereby influencing job burnout through the “job stress → work engagement” chain.

**Conclusions:**

This study extends the application of event systems theory and enriches the literature about how the COVID-19 pandemic impacted PHC medical staff job burnout. The findings derived from our study have critical implications for current and future emergency response and public policy in the long-term COVID-19 disease management period.

## Introduction

Since late 2019, a worldwide epidemic of Corona Virus Disease 2019 (COVID-19) has been occurring, causing considerable strain on healthcare systems. Although COVID-19 was declared not to be a Public Health Emergency of International Concern (PHEIC) by the World Health Organization in May 2023 [1], millions of people continue to be severely impacted by ongoing infection and re-infection, resulting in thousands of deaths each week [2]. Moreover, uncertainty remains regarding the possible emergence of new virus variants, which could potentially trigger fresh outbreaks [1]. Therefore, healthcare systems are required to take on a sustainable approach to COVID-19 infection prevention and control. However, during the past three years, healthcare workers have taken great efforts to rapidly control the epidemic with medical treatment and care, facing excessive workload and psychological stress, leading to a significant increase in the emergence of burnout [3, 4]. This negative psychological state of healthcare workers may continue or even develop in the post-epidemic era [5], which is not conducive to long-term sustained COVID-19 prevention, control and management efforts.

Job burnout is the response to prolonged work-related emotional, physical and mental stress, characterized by energy depletion or exhaustion, negative mood and reduced professional efficiency [6]. Empirical evidence has demonstrated that the medical profession is particularly susceptible to burnout because of the constant exposure to harsh environments such as mental and physical suffering or death [7, 8]. Especially in the context of the COVID-19 pandemic, health services surged in a short period, causing strong psychological and physiological stress on medical staff and increased burnout among medical staff. The most direct impact of job burnout on health care providers is job dissatisfaction, decreased productivity, and high staff turnover [9–11]. These negative consequences may further adversely affect the quality of patient care as well as the healthcare system’s ability to respond to public health emergencies [10, 12]. According to a meta-analysis conducted by Ghahramani and colleagues, the prevalence of job burnout among healthcare providers during the epidemic was 52%, which was significantly higher than the rates reported in the previous two decades, ranging from 32–34% [13]. Consequently, burnout in medicine has become an important public health issue that has attracted a great deal of attention from researchers and managers.

Over the past few years, many studies have been conducted on burnout among medical personnel in the context of COVID-19, however, most studies were conducted prior to the emergence of the Omicron variant. In 2022, China encountered a prevalence of the Omicron variant, resulting in another outbreak of COVID-19 [14]. Since March 2022, the epidemic in Jilin Province has expanded rapidly, characterized by multiple sporadic, clustered infected, and widespread [15]. Between March and November, the number of COVID-19 cases (including asymptomatic infections) in Jilin Province increased significantly to more than 87,000 cases, in contrast to less than 1,000 cases recorded in the province before March [16]. The surge in cases has imposed tremendous strain on society, especially the healthcare system. Compared with previous pandemics, COVID-19, especially the Omicron strain, has faster spread, more difficulty in prevention and control, and wider spread. These characteristics correspond to the attributes of event criticality, disruption and novelty [17]. In the COVID-19 pandemic, as the first line of defense, the primary health care (PHC) system confronts unprecedented challenges [18]. Although limited ability to act on the morbidity and mortality of severe cases, PHC can apply multiple interventions to reduce the infection spread and reduce the social and economic impact of social distancing measures [19]. During the COVID-19 pandemic globally, PHC takes on the additional responsibility of COVID-19 screening, referral, monitoring, education and publicity, and also needs to meet citizens’ medical care requirements as usual [20, 21]. In response to COVID-19 variants with higher transmissibility, China adopted a new strategy called “Dynamic COVID-zero” from August 2021, and PHC providers play an important role in halting the spread of the epidemic in the community [22]. However, the surge in cases has resulted in medical staff working under high-pressure, high-load, and high-risk circumstances for extended periods, creating conditions conducive to burnout [23, 24].

To date, numerous scholars have conducted research on the present condition and potential mechanisms of job burnout among medical personnel during the COVID-19 pandemic [7, 13, 23, 24]. However, the majority of studies utilize the pandemic as the research background and rarely directly explore the impact of the COVID-19 event itself on medical personnel burnout. Our study used event systems theory to evaluate COVID-19 event strength and to explore its impact on PHC medical staff job burnout as well as potential mechanisms during the Omicron variant outbreak. This research has important implications for current and future emergency response and public policy in the long-term COVID-19 disease management period.

## Theory framework and hypotheses

### Event system theory

According to event systems theory, the time, space, and strength of events occurring in life or work have dynamic effects on individuals’ psychological and behavioral responses [25]. To some extent, the strength of the event reflects the intensity of the event at a specific time and spatial, which conveys individuals’ perceptions of the significance and impact of the event. Event strength attributes include novelty, criticality and disruption [25]. Event strength novelty reflects the extent to which an event differs from current and past behaviors [25]. With the introduction of the highly transmitted variant into China, the management of the epidemic in China has shifted from a normalized stage of prevention and control to the “dynamic COVID-zero” stage, and the focus of epidemic prevention and control has changed from strict prevention of importation to efficient management of disseminated cases and aggregated outbreaks, thus, the work procedures and intensity of PHC personnel have also changed significantly [22, 26]. Therefore, the COVID-19 pandemic at this stage is relatively novel. Criticality reflects the degree to which an event is important or is a priority for organizations and individuals [25]. From the critical perspective, China’s " dynamic COVID-zero” strategy requires significant human and physical resource coordination and sustained efforts from PHC, making COVID-19 prevention and control an important, necessary and priority task [22]. Disruption reflects the degree to which an event changes the organization and individuals [27]. From the disruptive perspective, COVID-19 event subverts the daily work of PHC, and medical staff in PHC became the mainstay of nucleic acid testing, population screening, transport and reporting [28]. Therefore, the COVID-19 pandemic especially the Omicron variant outbreak in China has the three characteristics mentioned above.

To date, several recent studies have applied event systems theory or event strength to explore the possible effects of COVID-19 events on individuals and organizations. For example, Sheng F et al. investigated the effect of COVID-19 event strength on the entrepreneurial intentions of college students [29]; Zhou J explored the impact of COVID-19 pandemic strength on work fatigue among policemen [30]; and Deng H et al. examined the effects of COVID-19 event strength on employees’ turnover intentions [31]. Fewer studies, however, have used the event systems theory to investigate the impact of COVID-19 event strength on the mood and behavior of medical professionals, particularly in the PHC context. Due to the sudden nature and widespread impact of COVID-19, this pandemic has certainly posed considerable obstacles to the work and lifestyles of PHC medical professionals [32, 33]. Therefore, we chose COVID-19 event strength as the main variable in our study to assess its impact on job burnout among this cohort.

### Relationship between COVID-19 event strength and job burnout

According to event systems theory, the extent to which an event triggers controlled information processing and subsequent action depends on the assessment of its strength [25]. The higher strength of an event requires the entity more to adjust its mental and behavioral patterns as well as allocate more attention and resources to cope with the event [30]. According to the Job Demand-Resources (JD-R) model, excessive work demands and lack of resources for medical staff due to COVID-19 events can gradually deplete staff energy and result in burnout [34]. When medical professionals are long-term exposed to such work environments characterized by high-risk, high-emotional demands, high workload and job requirements, lack of relaxation activities and uncertain security, they face an increased risk of job burnout [7, 23]. Meanwhile, prolonged separation from family members and limited social support during the implementation of travel restrictions and quarantine requirements in response to the epidemic may lead to burnout among medical professionals [35, 36]. Furthermore, studies also have found that during the pandemic, health professionals and their relatives were also exposed to more violence and stigma, triggered or exacerbated professionals’ stress, anxiety and burnout [37, 38]. Therefore, the following hypothesis is proposed in this study.

#### Hypothesis 1

The COVID-19 event strength positively affects job burnout.

### The mediating role of job stress

Job stress is a response to an employee’s perception that their job demands exceed their ability to cope [39]. When this situation persists or is not managed properly, the impact of job stress can be detrimental to the employee, the organization, and society at large [40]. As front-line warriors in the prevention and management of the COVID-19 outbreak, medical personnel confront a significant risk of infection [41]. Numerous studies have demonstrated that the COVID-19 pandemic significantly increased the job stress of medical professionals [42–44]. The demand-control (JDC) model of Robert Karasek, which holds that job stress comes from the combined impacts of job demands and job control in the workplace, is one of the most significant and influential theoretical models of job stress management [45]. Specifically, the model argues that job-related stress arises when individuals experience high job demands and low job control simultaneously. Several studies have revealed that the ongoing COVID-19 pandemic imposes tremendous work demands and limited work control on medical personnel [46, 47]. Consequently, the COVID-19 event strength may heighten job stress among medical personnel, leading to adverse impacts on their health and well-being [43].

Prolonged exposure to work-related stress can lead to various adverse outcomes for medical professionals, particularly burnout syndrome [48, 49]. According to the multidimensional model of Maslach, job burnout is a sustained response to chronic stress [6]. PHC practitioners frequently experience a range of stressors, such as heavy workloads, shortages of skilled professionals, and lack of career advancement prospects, all of which can lead to burnout [50, 51]. Particularly during the COVID-19 pandemic, the workload, operating conditions, and task structure of PHC professionals changed significantly [52]. The high workload, unsafe workplace, and lack of training resulted in increased job stress [53, 54]. The study conducted by Molero et al. based on the JDC model also found that nurses’ work stress surged during the COVID-19 pandemic, and even led to job burnout [46]. Therefore, we presume that COVID-19 event strength may be able to influence PHC medical staff job burnout through job stress. In summary, we proposed Hypothesis 2:

#### Hypothesis 2

Job stress mediates the relationship between COVID-19 event strength and job burnout.

### The mediating role of work engagement

Work engagement is a positive, fulfilling, work-related state of mind characterized by vigor, dedication and absorption [55]. Vigor is characterized by high energy and mental resilience at work. Dedication is defined as an individual’s strong engagement in work and experiencing a sense of significance and enthusiasm. Finally, absorption is defined as the ability to concentrate and immerse in one’s work [56]. Work engagement of healthcare professionals is considered a positive psychological state that is associated with the delivery of high quality, cost-effective healthcare services [57]. According to the JD-R model, job resources are the main factor that motivates employees to participate in their jobs [34]. However, due to limited work resources, including physical resources (i.e., personal protective equipment) and human resources, the COVID-19 event presented a barrier to the engagement of health professionals [58]. In addition, event systems theory suggests that the stronger the event (the more novel, disruptive and critical), the less employees may not be able to drive their physical, cognitive and emotional energy into their job role performance as normal [25, 59]. The study conducted by Saadia et al. also found that fear of COVID-19 impaired employee engagement [60]. Therefore, we believe that the COVID-19 event strength may negatively affect PHC medical staff work engagement.

During the COVID-19 pandemic, the work engagement of PHC providers was highlighted because of its positive impact on the community from both a health and economic perspective [61]. Work engagement is often described as a useful coping skill for healthcare professionals and is seen as one of the key solutions to help prevent and recover from burnout [62]. One possible explanation for this could be that individuals who are more engaged in work have more positive emotions and feelings of satisfaction, which in turn alleviates or reduces energy expenditure and ultimately alleviate burnout [63]. Many studies have shown that there is a negative correlation between work engagement and job burnout. For example, Kusurkar et al.‘s study on PhD students in medicine showed that low engagement was associated with moderate and high burnout scores [64]. Similarly, López-Cabarcos et al. found that during the COVID-19 epidemic, nurses with higher work engagement experienced lower levels of burnout when confronted with elevated work requirements [65]. Combined with the above evidence, it is logical to assume that COVID-19 event strength may hinder the motivation of PHC providers to engage in their work, ultimately resulting in job burnout. Hence, this study hypothesizes the following:

#### Hypothesis 3

Work engagement is a mediating variable in the relationship between COVID-19 event strength and job burnout.

### The chain mediating role of job stress and work engagement

Healthcare professionals frequently encounter numerous job-related stressors that can have negative impacts on both their physical and mental health, as well as undermine work engagement and healthcare outcomes [51, 66]. The transactional model of stress emphasizes that an individual’s perception of stress degree directly leads to different emotional and behavioral reactions [67]. In other words, employees who experience high work stress may not be totally engaged in their work. A large number of studies conducted in healthcare settings had also demonstrated that job stress hinders the work engagement of healthcare professionals [51, 68, 69]. Following the outbreak of the COVID-19 pandemic, PHC medical staff encountered notable modifications, especially in terms of workload, job content, psychological stress, and infectious practice environment [32]. Consequently, the immense stress and increased workload caused by the epidemic may negatively affect the work engagement of PHC providers, which, in turn, may have resulted in significant job burnout. Thus, we pose Hypothesis 4:

#### Hypothesis 4

Job stress and work engagement have a chain mediating effect in the relationship between COVID-19 event strength and job burnout.

## Method

### Study design and participants

The research design used a cross-sectional survey. The quantitative study was conducted in Jilin Province, China during November 2022. This study was approved by the Ethics Committee of the School of Public Health, Jilin University(20210804). We used a multistage stratified convenience sampling method to select our study participants. In the first sampling stage, we selected six of the nine cities or autonomous prefectures in Jilin province based on the geographical distribution and economic characteristics. In the second stage, we selected 2 community healthcare centers and 6 township health centers from each city or autonomous prefecture, considering the proportion of the number of community healthcare centers and township health centers in Jilin Province. In the final sampling stage, we solicited the voluntary participation of medical staff from the selected primary healthcare institutions and conducted an independent self-reported questionnaire.

With the assistance of the Jilin Provincial Health Care Commission, the questionnaire was distributed using the Wenjuanxing online electronic survey system (https://www.wjx.cn/). All participants signed the informed consent form on the first page of the questionnaire and completed it via WeChat or webpage. The inclusion criteria for participants were as follows: (1) currently working full-time at the selected primary healthcare institutions; and (2) have participated in COVID-19 outbreak prevention and control within the previous year. The exclusion criteria was that the staff worked less than half one year. To ensure data quality, the same IP address can only be used once. A total of 1158 medical professionals completed the questionnaire, and after removing invalid responses, a final sample of 1148 was included for analysis. In this study, 27 items (11 COVID-19 event strength items, 9 work engagement items, 1 job stress item,1 job burnout item, and 5 covariates) were included. Benter and Chou indicated that the sample size should be 10 times the number of variables in the analysis [70]. Thus, the minimum sample size in our study was 270 which was considered sufficient to provide good statistical power.

## Measures

### COVID-19 event strength

COVID-19 event strength was assessed using the Chinese version of the Event Strength Scale developed by Morgeson [25] and translated into Chinese by Liu D et.al [71]. The scale has 11 items with three dimensions: event novelty, event criticality, and event disruption. A sample item is “COVID-19 event requires medical staff to make adjustments to previous work methods”. Each item was scored by 5-point Likert scale ranging from 1 (not at all) to 5 (very large extent). Items in the event novelty subscale were reverse scored. This Chinese version of the scale has been widely used to measure COVID-19 event intensity in previous Chinese studies and has been found to have good reliability [71–73]. In the present study, the Cronbach’s alpha coefficient of the scale and subscales ranged from 0.797 to 0.912.

### Work engagement

Work engagement was measured by the short version of the Utrecht Work Engagement Scale (UWES) [56]. The instrument consists of nine items that measure three dimensions of work engagement: vigor(three items), dedication (three items) and absorption (three items). Each item was scored by 7-point Likert scale ranging from 0 (never) to 6 (every day). Scores for all items were positive, so higher scores indicate higher levels of work engagement. In the present study, the Cronbach’s alpha coefficient of the scale and subscales ranged from 0.872 to 0.957.

### Job stress

One item we used to measure job stress was “How stressed do you feel about your job?“. Responses were given on a 5-point Likert-type scale ranging from (1) not at all stressful to (5) extremely stressful. Higher scores indicate greater levels of job stress. The measure has been widely used in job stress research [74–76].

### Job burnout

We measured burnout by the Dolan single-item measure which is reliable and validated among primary care staff [77]. The question asked “Overall, based on your definition of burnout, how would you rate your level of burnout?” The burnout questionnaire was scored from 1 (no burnout symptoms) to 5 (severe burnout symptoms).

### Covariates

The questionnaire included variables for possible confounding controls in the analysis of the association between COVID-19 event strength and job burnout. The variables evaluated were sex (female or male), age group (≤ 35 years; 36–45 years; ≥ 46 years), technical titles (no or primary title; intermediate title; vice-senior or senior title) workplace type(community healthcare centers or township health centers), and whether exposed to COVID-19 cases or suspected cases.

### Statistical analysis

Excel 2021 was used to complete the data conversion and entry, and IBM SPSS 24.0 software was used to analyze the data. As this study collected self-reported data, we tested the common method bias as recommended by Podsakoff et al [78]. Harman’s single-factor test was used to test for possible common method bias. The results show that a single factor contributes 37.58% in variance, which is less than 50% of the observed variance, indicating that our data were not affected by the common method bias. Descriptive analysis, independent t-test and one-way analysis of variance (ANOVA) were used to describe the medical staff’s sociodemographic characteristics and to compare the distribution of burnout, respectively. Pearson’s correlation analysis was used to analyze the correlation between COVID-19 event strength, job stress, work engagement, and job burnout. The mediation model was analyzed using SPSS PROCESS 3.5 macro. A chain-mediation model was tested using Model 6. Indirect effects were tested using 5000 bootstrap re-samples, with 95% confidence intervals (excluding zero) indicating significant effects. In addition, the model controlled for covariates (age, gender, technical titles, workplace type, and whether exposed to COVID-19 cases or suspected cases).

## Results

### Participant demographic characteristics and relationship with job burnout

A total of 1148 medical professionals participated in our study. The average age of the participants was 40.97 (SD = 9.25) years. The majority (80.49%) participants were female. 36.76% of the medical staff reported having been exposed to COVID-19 cases or suspected cases. Other demographic information is presented in Table 1.

**Table 1:** Differences in medical staff job burnout by demographic characteristics (N = 1148)

		n (%)	M ± SD	t/F	p
Age	≤ 35 yrs	365(31.79)	3.29 ± 1.20	9.600	< 0.001
	36–45 yrs	395(34.41)	3.09 ± 1.12		
	≥ 46 yrs	388(33.80)	2.93 ± 1.10		
Gender	Male	224(19.51)	3.08 ± 1.15	-0.353	0.724
	Female	924(80.49)	3.11 ± 1.15		
Technical titles	No or primary title	764(66.55)	3.08 ± 1.17	0.495	0.610
	Intermediate title	242(21.08)	3.17 ± 1.09		
	Vice-senior or senior title	142(12.37)	3.08 ± 1.11		
Workplace type	Community healthcare center	410(35.71)	3.25 ± 1.15	3.399	< 0.001
	Township health centers	738(64.29)	3.01 ± 1.13		
Whether exposed to COVID-19 cases	Yes	422(36.76)	3.26 ± 1.11	3.692	< 0.001
	No	726(63.24)	3.01 ± 1.16		

### Descriptive statistics and correlation analysis

The means, SDs, and correlations between the four key variables are shown in Table 2. COVID-19 event strength was positively correlated with job stress (r = 0.152, p < 0.01), job burnout (r = 0.175, p < 0.01), and negatively correlated with work engagement (r = -0.114, p < 0.01). Job stress was positively correlated with job burnout (r = 0.299, p < 0.01), and negatively correlated with work engagement (r = -0.257, p < 0.01). Work engagement was negatively correlated with job burnout (r = -0.255, p < 0.01).

**Table 2:** Correlations among variables, means, and standard deviations

	M ± SD	1	2	3	4
1. COVID-19 event strength	36.78 ± 3.46	1			
2. Job stress	3.90 ± 1.07	0.152^**^	1		
3. Work engagement	31.90 ± 12.45	-0.114^**^	-0.257^**^	1	
4. Job burnout	3.10 ± 1.15	0.175^**^	0.299^**^	-0.255^**^	1

Notes: ** means that p-value is < 0.01

### Chain mediation model analysis

The study used model 6 in PROCESS macro of SPSS [79] to examine the chain mediating effects of job stress and work engagement between COVID-19 event strength and job burnout with gender, age, technical title, workplace type, and whether exposure to COVID-19 cases as covariates. Table 3 shows the results of the regression analysis. The results show that in model 1, COVID-19 event strength had a significant positive predictive effect on job burnout (β = 0.163, p < 0.001), therefore H1 was supported. In model 2, COVID-19 event strength significantly positively predicted job stress (β = 0.151, p < 0.001). In model 3, COVID-19 event strength significantly negatively predicted work engagement (β=-0.067, p < 0.05) and job stress significantly negatively predicted work engagement (β=-0.249, p < 0.001). In model 4, COVID-19 event strength (β = 0.110, p < 0.001), job stress (β = 0.238, p < 0.001) significantly positively predicted job burnout, while work engagement significantly negatively predicted job burnout (β=-0.165, p < 0.001).

**Table 3:** Regression analysis of variable relationships in the model

	Regression Equation		Overall Fit Index			Significance of Regression Coefficient		
	Result Variable	Predictive Variable	R	R^2^	F	β	SE	t
Model 1	Job burnout	COVID-19 event strength	0.261	0.068	13.900^***^	0.163	0.010	5.692^***^
Model 2	Job stress	COVID-19 event strength	0.202	0.041	8.049^***^	0.151	0.009	5.193^***^
Model 3	Work engagement	COVID-19 event strength	0.322	0.104	18.814^***^	-0.067	0.102	-2.361^*^
		Job stress				-0.249	0.335	-8.713^***^
Model 4	Job burnout	COVID-19 event strength	0.409	0.167	28.561^***^	0.110	0.009	3.997^***^
		Job stress				0.238	0.031	8.330^***^
		Work engagement				-0.165	0.003	-5.783^***^

Note: * means that p-value is < 0.05; *** means that p-value is < 0.001

**Fig. 1:**
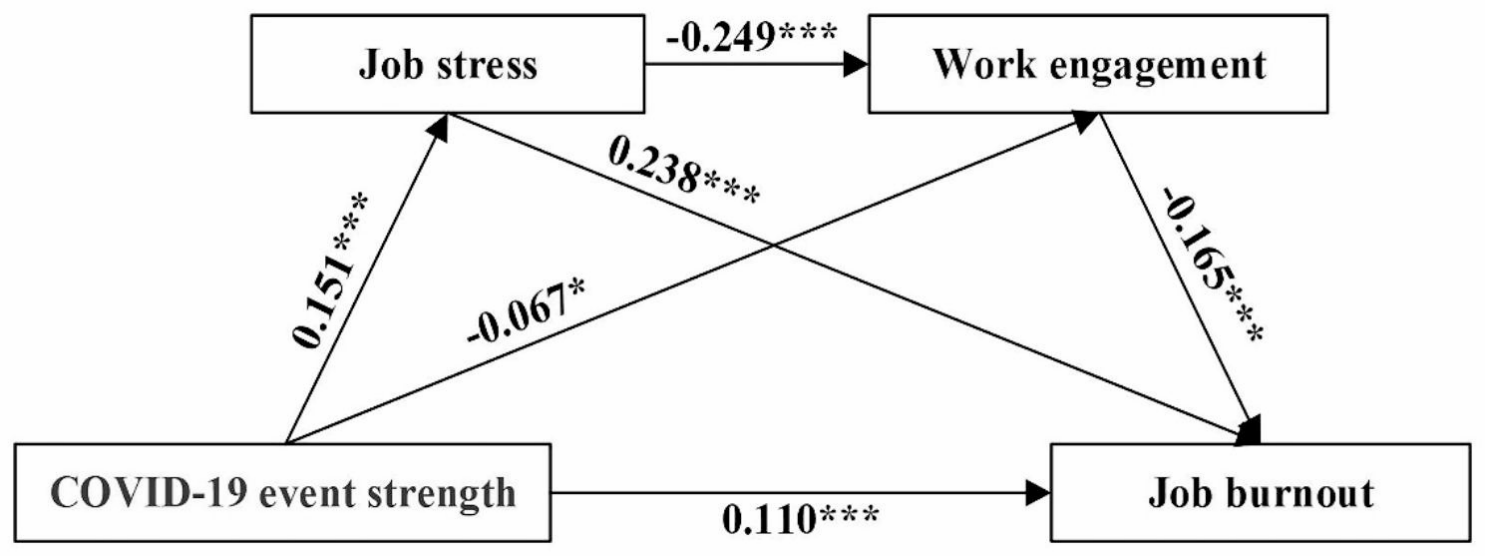
Mediation model of job stress and work engagement between COVID-19 event strength and job burnout Note: All path coefficients showed in the figure were standardized coefficients. * means that p-value is < 0.05; *** means that p-value is < 0.001

Table 4 shows the results of the analysis of the chain mediation model using the bootstrapping method. Figure 1 shows the path diagram and effect values of the chain mediation model. As shown in Table 4, job stress and work engagement mediated the effect of COVID-19 event strength on job burnout with a mediated effect value of 0.053, accounting for 32.52% of the total effect. The total indirect effect consisted of three pathways of indirect effects. Path 1 consisted of COVID-19 event strength→ job stress→ job burnout, with a confidence interval that did not contain 0 ([0.021, 0.052]), indicating that the indirect effect generated by this path was significant. Path 2 consisted of COVID-19 event strength→ work engagement→ job burnout, with a confidence interval that did not contain 0 ([0.001, 0.025]), indicating that the indirect effect generated by this path was significant. Path 3 consisted of COVID-19 event strength→ job stress→ work engagement→ job burnout, with a confidence interval that did not contain 0 ([0.002, 0.011]), indicating that the indirect effect generated by this path was significant. The results indicated that the effect of COVID-19 event strength on job burnout was achieved through the chain mediating effect of job stress and work engagement as well as the separate mediating effects of each. Thus, H2–H4 of this study are confirmed.

**Table 4:** Chain-mediated model effect tests for job stress and work engagement

	Effect Value	Boot SE	Bootstrap 95% CI		Proportion of Relative Effect
			Boot LLCI	Boot ULCI	
Total effect	0.163	0.029	0.107	0.219	100%
Direct effect	0.110	0.028	0.056	0.164	67.48%
Total indirect effect	0.053	0.013	0.032	0.077	32.52%
Path 1	0.036	0.008	0.021	0.052	22.09%
Path 2	0.011	0.006	0.001	0.025	6.75%
Path 3	0.006	0.002	0.002	0.011	3.68%

Note: Boot SE, Boot LLCI, and Boot ULCI refer to the standard errors and lower and upper 95% confidence intervals of the indirect effects estimated by the bias-corrected percentile Bootstrap method, respectively. Path 1: COVID-19 event strength → job stress → job burnout; path 2: COVID-19 event strength →work engagement →job burnout; path 3:COVID-19 event strength → job stress →work engagement →job burnout

## Discussion

In this study, we investigated and analyzed job burnout among PHC medical staff during the epidemic of the COVID-19 omicron variant in 2022. Then, we explored the relationship between COVID-19 event strength and job burnout, focusing on elucidating the underlying mechanisms. The results indicated that COVID-19 event strength not only directly influenced job burnout, but also indirectly through the mediation of job stress and work engagement. We have made some theoretical and practical contributions to the COVID-19 literature by correlating individual differences in the strength of impact of the COVID-19 epidemic directly with the negative consequences for PHC medical staff.

### Theoretical implications

This research contributes to the study of COVID-19 events in several ways, furthering our understanding of the ripple effects of this epidemic on the healthcare sector. First, our study examined the impact of COVID-19 event on job burnout in PHC medical professionals, enriching the field of research on the impact of public health events. Despite receiving significant attention, the COVID-19 pandemic has been largely overlooked in existing studies in terms of its event attributes. Our study introduces the event systems theory, a fundamental theory of organizational behavior, into the field of PHC and adopts the concept of event strength to directly evaluate the individual differences in the degree of impact of COVID-19. Based on the event systems theory and a focus on the psychological aspects of the epidemic, we argue that the COVID-19 pandemic is a work-related event characterized by employees’ perceptions of the event’s novelty, disruption, and criticality and was found to directly lead to PHC medical professional job burnout. This conclusion is similar to Zhou J’s study based on event systems theory, which found that COVID-19 events lead to increased police work fatigue [30]. In conclusion, our study enriches the research of the event system theory and expands its applications.

Second, our study discovered that job stress was an important mediator in explaining how the COVID-19 event strength contributed to job burnout among PHC medical professionals. Unlike intra-organizational factors, traumatic events such as the COVID-19 pandemic are external shocks beyond the control of the organization and its employees [80]. Our research extends the study of job stress by looking beyond the widely studied intra-organizational stressors and examining the negative effects of acute extra-organizational stressful events. In fact, PHC practitioners have been exposed to oppressive work environments since the COVID-19 outbreak. Since February 2022, the most severe COVID-19 outbreak in China driven by the Omicron variant, has led to successive lockdowns in Shenzhen City, Jilin Province, Shanghai Municipality, and other areas [81]. During this challenging period, PHC institutions continuously faced the high stress of rapid pressure of the Omicron variant, and the workload and infection risk of PHC medical professionals were significantly different compared to the regular prevention and control period, which may result in a dramatic increase of job stress [20]. Many studies conducted in China have reported a significant association between COVID-19-induced stress and negative psychological states [82–84]. Our finding is similar to these studies but complement the literature after the rapid spread of the Omicron variant, revealing the negative impact of the COVID-19 pandemic, a public health emergency, on the mental health of PHC providers at different periods.

Finally, this study proposed and tested the effect of work engagement as a mediator of the relationship between COVID-19 event strength and job burnout, and revealed a chain mediating effect of job stress and work engagement. Similar to our findings, several studies have found that job stress impairs work engagement in medical professionals [51, 85]. However, Van et al.‘s study found that nurses’ work engagement counteracted work-related stress reactions, and that workload did not necessarily affect work engagement [86]. Challenging work may also produce beneficial outcomes, such as greater work engagement through feelings of accomplishment and a sense of pride [87]. But our findings demonstrated that the work stress accompanying the COVID-19 event seemed to exceed the coping capacity of PHC providers. It also indicates that the impact of public health emergencies is different from the stress in ordinary work environments. Furthermore, during the COVID-19 pandemic, PHC medical professionals frequently conducted repetitive procedures such as nucleic acid screening and isolation management, which can also cause job stress and reduce work engagement [20, 88]. Despite these findings, work engagement among PHC medical professionals in this study remained at higher levels presumably indicating the self-giving nature of PHC medical professionals when public health is threatened by infectious diseases.

### Practical implications

Our findings have several practical implications. First, considering the negative effects of COVID-19 event strength, PHC managers can apply event systems theory to design training and intervention programs aimed at mitigating the perceived strength of the COVID-19 crisis by eliminating novel, disruptive, and critical perceptions of the crisis among medical staff [25]. For example, PHC can design clear and understandable work procedures and guidelines during the epidemic to reduce the perceived novelty of the COVID-19 epidemic’s novelty. Furthermore, although the COVID-19 pandemic may be coming to an end, the epidemic’s psychological impact may persist longer than the disease itself [5]. Therefore, PHC administrators should constantly implement strategies to lessen the psychological load of health professionals, including establishing psychological intervention teams and organizing psychological support programs or activities, etc [89].

As demonstrated by this research, job stress and work engagement are important mechanisms by which COVID-19 events affect PHC medical staff burnout. PHC administrators should continuously monitor employee stress and work engagement and intervene timely, especially during public health emergencies (e.g., COVID-19 pandemic). At the organizational level, PHC institutions should provide adequate physical facilities, a supportive health climate, and benefit incentives that can help medical staff reduce their perceived stress and provide sufficient work resources to encourage them to actively participate in their work [90]. At the medical staff level, Managers should focus on stimulating the potential positive attributes of medical staff and encouraging them to adopt positive coping styles to manage stress [91]. In summary, administrators need to focus on creating and maintaining a high-quality primary care workplace, which is critical to reducing medical professional stress, improving work engagement, reducing burnout, and ensuring the sustainability of the health system [92].

### Limitations and future research

Despite the valuable findings of the present study, some limitations should be highlighted in order to improve future research. First, all participants were from Jilin province, which is one of the regions in China that was most severely affected by COVID-19 in 2022. Therefore, the study results may not be fully applicable to PHC medical professionals in other regions of China. It is recommended that the sample scope should be expanded in future studies to further examine cross-regional differences. Secondly, our study was based on a cross-sectional design and causal inference was limited. Future studies may use longitudinal designs to obtain stronger empirical evidence of causality. Finally, the impact of the COVID-19 pandemic was measured using event strength in our investigation. However, event systems theory also focuses on explaining the impact of events on organizations and individuals in terms of temporal and spatial attributes [25]. Subsequent studies may enlarge the event dimension to measure the epidemic’s temporal and spatial attributes and delve deeply into its influence on the PHC system.

## Conclusions

Based on event systems theory, this study examined the effect of the COVID-19 event strength on job burnout in PHC medical professionals and the underlying psychological mechanisms between the relationships. The results of the study showed that COVID-19 event strength had a significant positive predictive effect on job burnout among medical professionals. Job stress and work engagement played both individual mediators and chain mediators between COVID-19 event strength and job burnout. Considering the negative impact of COVID-19 events, PHC managers can apply event systems theory to design training and intervention programs that can more efficiently tackle the challenges caused by public health emergencies. Meanwhile, PHC institutions should provide adequate work resources and stimulate potentially positive attributes of medical staff to help them manage stress and improve work engagement, subsequently reducing the incidence of burnout.

## Data Availability

The data and materials of the present study are available from the corresponding author on reasonable request.

## Abbreviations

COVID-19: Corona Virus Disease 2019
JD-R: Job Demand-Resources
JDC: Demand-control
PHC: Primary health care
PHEIC: Public Health Emergency of International Concern
UWES: The Utrecht Work Engagement Scale
